# Falling for a Balance Partner

**DOI:** 10.3389/fpubh.2014.00130

**Published:** 2015-04-27

**Authors:** Sara B. May, Cherie A. Rosemond

**Affiliations:** ^1^The University of North Carolina at Chapel Hill, Chapel Hill, NC, USA

**Keywords:** falling accident, fall prevention strategies, balance partner, older adults, opinion

The growing accumulation of knowledge about fall prevention strategies primarily reflects a research perspective where intervention data are rigorously collected and analyzed. However, the voices of program deliverers or participants who are part of these interventions are often aggregated and thus muted. With recognition of the growing importance of patient-centered care, we wanted to provide a personal reflection on the Balance Partner program, a CDC funded project to train peer leaders in fall prevention. In the story below, Sara was trained as a Balance Partner using a curriculum developed at The University of North Carolina’s Center for Health Promotion and Disease Prevention.

The Balance Partner Program provides training and support to community volunteers who are paired with a peer who is at risk for falls. Together, Balance Partners plan strategies to decrease the likelihood of a fall – strategies that could include joining a balance exercise program, improving home safety, or getting a vision check-up. By addressing social and emotional factors alongside knowledge about falls, the Balance Partner Program aims to increase older adults’ overall adherence to fall prevention activities above the 50% rate reported in literature (1). Sara, a 69-year-old volunteer, was paired with Georgia, who screened at high risk for falls during a Building Better Balance Screening in Asheville, NC, USA. As of this writing, Sara and Georgia had worked together for almost 6 months to implement strategies to reduce their falls risk.

## A Blossoming Partnership and Friendship

“How did we get together?” the smiling 81-year-old demure-looking lady asked me during my Saturday visit in her Asheville apartment. Now prone to memory lapses, Georgia had forgotten that she had difficulty in completing the balance tests at the Building Better Balance Screening offered in her apartment complex by the Land of Sky Area Agency on Aging. Just a few months before Georgia went through her balance screening, I had the opportunity to attend Balance Partner training (on my birthday) sponsored by UNC’s Center for Health Promotion and Disease Prevention. As it turned out, just when I was all set to find a fellow senior in need of volunteer assistance, Georgia was eager to reduce her risk of falling. We had a match!

Georgia and I started meeting in her apartment once or twice a week. Since she had moved into the apartment 3 years earlier, three tumbles had undermined Georgia’s confidence that she could avoid falling. She had walked with a cane for several years before needing the greater support of a walker. I, too, had three falls at home. Thankfully, I was blessed with only bruises to show for the experiences. I was motivated to help others stay upright after learning fall prevention techniques during physical therapy and implementing them around my home. I shared some of what I learned with Georgia.

We outfitted Georgia with a night-light in her bathroom and two lightweight flashlights to help find her way around her apartment when the electricity goes out. She now keeps her apartment clutter-free, avoiding throw rugs in which her walker might get tangled. I brought Georgia a monthly wall calendar to help her keep track of upcoming doctor’s appointments and I drive her to them as often as my schedule and health permits. As a bonus, I get more exercise by helping Georgia in and out of the car. She is only up for getting out when the weather is warm and the skies are sunny, which suits me fine. We are working with her physicians to find ways that Georgia might be able to lessen the number of trips to the bathroom during the night, thereby lessening the chances of her taking another tumble.

Although we are from diverse backgrounds and cultures, we easily settled in to focus on our similarities, including having several chronic health conditions and a desire to stay the healthiest we could for as long as possible. Georgia had given up taking baths, as she was unable to get up and out of the tub, even with grab bars on two sides of it. I have had railings installed on short stairways on my porches and learned to sit down when putting on my shoes to avoid losing my balance while standing on one foot. “I’m more aware of possible fall causes now and am very careful using my walker,” Georgia says. She adds, “Sara has suggested I change to a closer pharmacy so it’s easier to get my medications picked up. When Sara takes me shopping, she does the legwork for me, bringing me options of the items I’m looking for so I can save my energy for getting to and from her car.”

Isolation is one of the most difficult issues that Georgia has had to face since she had to give up her driver’s license last year. When I was asked to substitute as a co-facilitator for a January session of a Living Healthy with Chronic Conditions workshop at Georgia’s apartment complex, I persuaded her to come along and try it, suggesting that she sits close to the door since she was concerned that she might need to leave in a hurry to go to the bathroom. “I’m doing this for you,” she told me when I walked with her to the first class, but attending the rest of them has been for her. She loved the opportunity to interact with other residents in a small-group setting, especially since she is hard of hearing. She has kept returning to subsequent classes whether I am there co-facilitating or not. In fact, that is where she found her hall-walking buddies!

“I felt like it would help motivate me to have a Balance Partner,” Georgia said. “I want to stay out of a wheelchair and eventually go back to walking with only a cane.” Little did we anticipate at our first meeting that we would become fast friends. Georgia advised me on a good hairstyle to make my hearing aid less visible to others, and I helped her pick out a new wig, which makes Georgia look picture-perfect. Our outing to the wig shop even resulted in her getting to relive old times with the wig shop owner. When Georgia calls me to see how I am doing when she knows that I am sick and could use some cheering up, it feels like the frosting on the cake of our relationship. Even though she prefers salty to sugary foods and I am the opposite, we continue to enrich each other’s lives as we explore this “not for sissies” thing called aging in place.

## Conclusion

Helping others is often the pathway to helping oneself. Current research on community fall prevention interventions suggests that social and emotional factors such as isolation and boredom partially explain low adherence rates (2). Making new friends, sharing talents, and having fun are rarely reported in traditional research studies. This reflection reveals a personal account of why a falls prevention intervention effort based on supportive interactions can be so powerful.

## Conflict of Interest Statement

The authors declare that the research was conducted in the absence of any commercial or financial relationships that could be construed as a potential conflict of interest.

This paper is included in the Research Topic, “Evidence-Based Programming for Older Adults.” This Research Topic received partial funding from multiple government and private organizations/agencies; however, the views, findings, and conclusions in these articles are those of the authors and do not necessarily represent the official position of these organizations/agencies. All papers published in the Research Topic received peer review from members of the Frontiers in Public Health (Public Health Education and Promotion section) panel of Review Editors. Because this Research Topic represents work closely associated with a nationwide evidence-based movement in the US, many of the authors and/or Review Editors may have worked together previously in some fashion. Review Editors were purposively selected based on their expertise with evaluation and/or evidence-based programming for older adults. Review Editors were independent of named authors on any given article published in this volume.
